# Recent Evidence Regarding the Association Between Migraine and Suicidal Behaviors: A Systematic Review

**DOI:** 10.3389/fneur.2020.00490

**Published:** 2020-06-23

**Authors:** Leila Karimi, Dimi Hoppe, Christine Burdick, Melissa Buultjens, Tissa Wijeratne, Sheila G. Crewther

**Affiliations:** ^1^School of Psychology and Public Health, La Trobe University, Melbourne, VIC, Australia; ^2^Faculty of Social and Political Sciences, Ivane Javakhishvili Tbilisi State University, Tbilisi, Georgia; ^3^St. Vincent's Hospital Melbourne, Fitzroy, VIC, Australia; ^4^Department of Neurology, Western Health, AIMSS, Level Three, WHCRE, Sunshine Hospital, University Melbourne, St. Albans, VIC, Australia; ^5^Department of Medicine, Faculty of Medicine, University of Rajarata, Anuradhapura, Sri Lanka

**Keywords:** migraine, migraineurs, suicidality, suicidal behaviors, suicide, suicide attempt, suicide ideation, systematic review

## Abstract

**Objective:** The review presents a systematic analysis of literature investigating the association between migraine and suicidal behaviors.

**Introduction:** Migraine is a common neurological disorder. The prevalence of migraines increases with age from adolescence to adulthood in both sexes, and results in a substantial loss of productivity due to missing days of school or work and need for bed rest. Literature prior to 2015 suggests that migraine is a predictor of suicide. Given the worldwide public health interest in suicide prevention, we examined the literature collected from diverse, predominantly non-European, populations post-2015.

**Methods:** The databases used in this systematic review included: Medline, PsycINFO, EMBASE (Ovid), Science Direct (Elsevier), Cochrane, and PubMed for all available years of publication from January 2015 onwards. The review included participants aged 16 and over who had been diagnosed with migraines with the following outcome variables: any suicidality, both fatal and non-fatal; suicidal ideation; and suicidal behavior.

**Results:** The database searches yielded a total of 542 citations. Following title and abstract screening, 460 articles were excluded and a total of 21 citations were evaluated. After full-text review and excluding a further 11 non-eligible studies, a total of 10 studies were eligible for inclusion in the systematic review.

**Conclusions:** Current existing research highlights the important association between the increased risk of suicidal behaviors in the clinical and general population among chronic migraineurs with/without aura worldwide. Future studies are needed to facilitate the development of clinical guidelines for risk assessment, targeted interventions, and evidence-based treatment of migraine to reduce the risk of suicide among this vulnerable population.

## Introduction

Migraine is a neurological disorder with a prevalence rate of between 11 and 23% (1–5). Figures from the Global Burden of Disease Study (GBD) 2016 (3) highlight the detrimental effect of headache disorders, indicating migraine as a cause for 5.6% of disabilities worldwide (3, 4). According to the World Health Organization(WHO) (6), headache disorders are a worldwide public health problem that impose a major burden that negatively impact on family, social life, and employment. From a medical perspective migraine is the most prevalent, most disabling headache disorder, with frequent visits to the ER and doctors. Tension Type Headaches (TTH), meanwhile, are as common in the community but are less likely to result in visits to doctors as the headache disorder responds well to simple analgesics (7). According to WHO, about one third of people with headaches are also diagnosed with migraine (6).

Migraine is a recurrent headache disorder of 4–72 h duration and is predominantly associated with autonomic nervous system symptoms. Frequent migraine episodes are classified as chronic when they occur: (a) for a period of at least 3 months, (b) on more than 15 days per month, and (c) the headaches have migraine characteristics on more than half of the episodes (2). The prevalence of migraine increases with age from adolescence to adulthood, resulting in a substantial loss of productivity due to sick days, and is one of the main causes of disability globally. Indeed, 86% of migraine sufferers are of working age (5, 7).

The World Health Organization reports clinical anxiety and depression to be significantly more common in people with migraine vs. non-migraineurs (6, 8). Nović et al. (8) led a systematic review of the literature appearing between 1966 and 2014 and synthesized the evidence of suicidality including suicidal ideation (thoughts about suicide) and suicide behaviors (the suicide attempt itself), both fatal and non-fatal, and revealed a risk of suicidal behaviors in both clinical and non-clinical migraine populations. Some of the studies reviewed demonstrated that migraine was a predictor of suicidal behaviors even after controlling for psychiatric conditions (8). Similarly, a recent systematic review and meta-analysis (9) that investigated the relationship of migraine and suicidal ideation observed that migraine was a significant risk factor. The authors reported similar results even after some psychiatric comorbidities were considered (9). Statistics also demonstrated that suicide among children is rare while the highest rates were observed in mid-age adults (10).

Thus, in the present study we conducted a systematic analysis of the literature from 2015 to November 2019, investigating migraine specifically, as the most prevalent and debilitating headache type, and exploring its link with suicidal behaviors among adult participants of 16 years and older. This includes an analysis of previously unstudied populations in Asia, South America, and Ethiopia.

## Methods

The Joanna Briggs Institute guidelines on etiology and risk (11, 12) and the Preferred Reporting Items for PRISMA (13) were used in this systematic review. It was registered with Prospero (registration number CRD42020158903).

### Search Strategy

A three-step search procedure was undertaken (11, 12). A preliminary search of the databases of Medline, PsycINFO, EMBASE (Ovid), Science Direct (Elsevier), Cochrane, and PubMed were taken as a first step. The second step included a more comprehensive and focused search of all the keywords. Finally, a manual search of the main web browsers was undertaken as reported by Moola et al. (11).

### Types of Studies Included

The systematic review considered all quantitative study designs including observational/cohort studies and randomized controlled trials of persons with medically diagnosed migraine aged 16 and over. Only English language papers were considered in the review due to time constraints and limited resources to interpret other languages. Studies published from January 2015 to November 2019 were included. This date range was selected in order to retrieve and investigate all studies that had not been considered in previously conducted systematic reviews, including Nović et al. (8).

### Information Sources

A systematic search was conducted on 30 November 2019 using Medline, PsycINFO, EMBASE (Ovid), Science Direct (Elsevier), Cochrane, and PubMed for all available years of publication from 2015 onwards. The following key terms were used: (a) Migraine/or chronic or tension or intractable/or headache, Migraine disorders/or Tension type, headache/or Headache disorders; or (b) Photophobia/Aphasia/facial nerve/or oculomotor nerve/or exp vasomotor system/sensory adj2 (sensitivit^*^ or overload or anomal/Autonomic nervous system or ANS/Transient or temporary) adj2 (Hemiparesis or Speech difficult Facial or oculomotor Vasomotor system/Light sensitivit^*^ or photophobi Light sensitivit^*^ or photophobia; or a or b; and (c) suicide/or suicidal ideation/or suicide, attempted/ (d) Self-Mutilation/ (e) self-harm or injurious behavior mutilation or injury or destruction or killing; or c/d/e. In order to center the review on peer reviewed articles gray literature was excluded.

## Inclusion/Exclusion Criteria

### Participants

The review included studies whose participants were older than 16 years who were diagnosed with migraine. Studies were excluded if they included participants younger than 16 years old. Patients were required to experience at least one severe migraine episode per month or more (14).

### Study Selection

**Figure 1:**
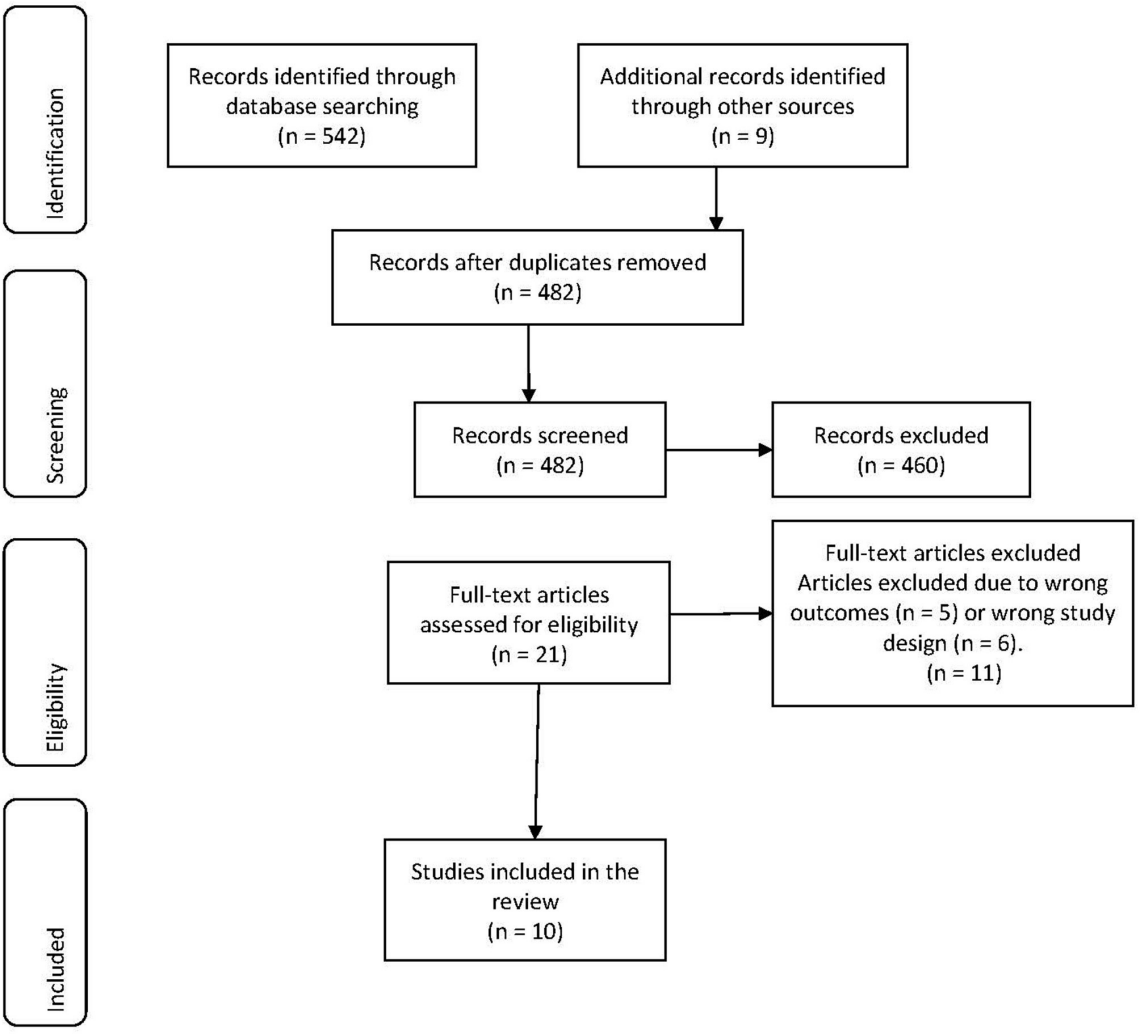
PRISMA chart: search results for migraine and suicidal behaviors.

All the identified articles were imported to EndNote 9 and duplicate citations were removed then imported to Covidence System (Covidence.org) for further screening. The abstracts were screened by two reviewers independently (MB, CB) followed by the full text of the included citations. Conflict opinions were resolved by a third reviewer (LK). The search results for article selection are presented in Figure 1.

### Data Extraction

Two reviewers (MB, CB) extracted the data based on the standardized data extraction tools in Covidence. A summary of the included articles is detailed in Table 1.

**Table 1:** Summary of characteristics of included studies.

**Reference title First Author/Country of origin**	**Year of publication**	**Sample/** **Participant age**	**Study design/** **Study duration**	**Clinical diagnosis of migraine**	**Main findings**	Quality score^*^
Suicidal ideation in persons with neurological conditions: prevalence, associations, and validation of the PHQ-9 for suicidal ideation Altura KC Canada	2016	*n =* 208 Mean age of participants: 43.4 (range 18.1–75.1)	Prospective cohort study Participants were recruited from four outpatient clinics (Epilepsy Clinic, MS Clinic, Headache Clinic, and Stroke Clinic)—Patients had to be: 18 years of age or older; (b) fluent in English; (c) free of hearing impairment because they had to complete a telephone interview; and (d) free of physician-diagnosed moderate or severe dementia, moderate or severe developmental delay, or aphasia August 2012 to September 2013	Determination of migraine diagnosis was not stated in the study design. It was assumed patients had a pre-existing diagnosis of migraine as they were recruited from various neurological outpatient clinics	Overall, factors most strongly associated with suicidal ideation were depression, migraine, and anxiety The primary aim of the study was to validate the Patient Health Questionnaire (PHQ)-9 as a screening tool for suicidal ideation According to the PHQ-9, the 2-week point prevalence of suicidal ideation for migraine was 15.9% and on the structured clinical interview for DSM-IV was 12%. The PHQ-9 had good sensitivity for migraine (75%)	I = 0 II = 1 III = 1 IV = 0 V = 1 Total score = 3
B. Association between migraine and suicidal behavior among Ethiopian adults Berhane HY Ethiopia	2018	*n =* 1,060 Age 35.28 (*SD* 12.05)	Cross-sectional study Eligible participants included all adults attending the outpatient facility at the Saint Paul Hospital in Addis Ababa, Ethiopia and were patients evaluated in the internal medicine, general surgery, and gynecological outpatient departments December to July 2011	Trained nurses used an interview format to administer structured questionnaires A structured migraine assessment questionnaire adapted from a previously validated tool was used to classify migraine according to the ICHD-II criteria The Composite International Diagnostic interview (CIDI) was employed to assess depression and suicidal behaviors that were classified as ideation, plans, and attempts based on self-report	Migraine is associated with increased odds of suicidal behavior in the population of urban-dwelling Ethiopian adults The presence of migraine was associated with a 2.91-fold increased risk of suicidal behavior (OR: 2.91, 95% CI: 2.06–4.12) compared with participants without migraine (2.71-times after adjusting for confounders)	I = 0 II = 1 III = 2 IV = 0 V =1 Total score = 4
C. Association between lifetime headache and history of suicide attempts in the elderly Calati R France	2017	*n =* 1,965 Age 72.27 (*SD* 4.76)	Prospective cohort study Eligible participants were community-dwelling individuals randomly selected from the 15 electoral rolls of the Montpellier district who were aged 65 years and above March 1999 to February 2001	A neurologist assessed headache cases based on the International Headache Society guidelines	Lifetime headache was associated with lifetime suicide attempts (OR: 1.92, 95% CI: 1.17–3.15)	I = 0 II = 1 III = 0 IV = 0 V = 1 Total score = 2
D. Association of migraine headaches with suicidal ideation among pregnant women in Lima, Peru Friedman LE Peru	2016	*n =* 3,323 Age 28.2 (*SD* 6.3)	Cross-sectional study A cross-sectional study was conducted among pregnant women attending prenatal care clinics in Lima, Peru February 2012 to March 2014	Trained interviewers classified migraine using a questionnaire administered during early pregnancy Migraine classification (including migraine and probable migraine) was based on the International Classification of Headache Disorders (ICHD)-III beta criteria Suicidal ideation and depression were assessed using the Patient Health Questionnaire-9 (PHQ-9) scale during early pregnancy	Participants with migraine or probable migraine had more than a 2-fold increased risk of suicidal ideation (OR = 2.17; 95% CI: 1.80–2.61) compared with non-migraineurs. After adjusting for confounders, there was still an almost 2-fold increase in suicidal ideation (OR = 1.99; 95% CI: 1.64–2.41)	I = 0 II = 1 III = 1 IV = 0 V = 1 Total score = 3
E. Association between migraine and suicidal behaviors: a nationwide study in the USA Friedman, LE United States of America	2018	*n =* 156,172,826 Mean age 47	Cross-sectional study The Nationwide Inpatient Sample of hospitalisations compiled from USA billing data was analyzed Migraine, suicidal behaviors, and psychiatric disorders were identified based on the International Classification of Diseases, 9th Revision, Clinical Modification (ICD-9-M) diagnosis codes from hospitalization discharges (2007–2012) Discharge data from 2007 to 2012	Adult onset migraine diagnosis was based on the ICD-9-CM diagnosis codes	Individuals with migraine had a 2.07-fold increased risk of suicidal behaviors (OR = 2.07; 95% CI: 1.96–2.19) compared with non-migraineurs	I = 1 II = 1 III = 2 IV = 0 V = 1 Total score = 5
F. Suicide attempts among those with migraine: findings from a nationally representative Canadian study Fuller-Thomson E Canada	2019	*n =* 21,744 (with migraine *n =* 2,223) Mean age 85	Cross-sectional study This study was a nationally representative analysis of the 2012 Canadian Community Health Survey—Mental Health (CCHS-MH)	Participants were asked if they had been diagnosed with migraine headaches by a health professional (expected to last or have already lasted 6 months or more)	Individuals with migraine had an almost 3-fold higher prevalence of attempting suicide than those without migraine (males 7.5% vs. 1.9% and females 9.3% vs. 2.7%) OR = 3.40; 95% CI: 2.84–4.07	I = 1 II = 1 III = 2 IV = 0 V = 1 Total score = 5
G. Risk and predisposing factors for suicide attempts in patients with migraine and status migrainosus: a nationwide population-based study Harnod T Taiwan	2018	*n =* 13,605 (status migrainosus cohort) *n =* 21,485 (regular migraine cohort) *n =* 54,379 (comparison cohort) Age 45.7 (*SD* 14.8) (status migrainosus cohort) 45.6 (*SD* 15.1) (comparison cohort)	Cross-sectional study An analysis was conducted of a subset of the National Health Insurance Research Database of Taiwan and enrolled patients (20 years of age and older) who had ever received a diagnosis of regular migraines (RM) or status migrainosus (SM) between 2000 and 2012 in the RM and SM cohort January 2000 to December 2012	Migraine diagnosis was based on the ICD-9-CM diagnosis codes for RM 346 excluding 346.9 and SM 346.9 excluding 346.90 and 346.91	The status migrainosus cohort had a 1.81-fold risk of attempting suicide (OR = 1.81; 95% CI: 1.14–2.89) compared with the comparison cohort	I = 1 II = 1 III = 2 IV = 0 V = 1 Total score = 5
H. Association of suicide risk with headache frequency among migraine patients with and without aura Lin YK Taiwan	2019	*n =* 528 Age 33.7 (*SD* 10.3)	Cross-sectional study This cross-sectional study included 528 consecutive patients aged between 20 and 60 years attending a headache clinic at the Department of Neurology of the Tri-Service General Hospital (TSGH) in Taipei, Taiwan Patients with migraine, both with and without aura, were analyzed June 2015 to May 2017	Patients completed a screening questionnaire and were subsequently interviewed by a board-certified neurologist and headache specialist to make a diagnosis according to the International Classification of Headache Disorders, 3rd edition (ICHD-3 beta) Patients with migraine were determined to be with or without aura, based on the criteria of the International Headache Society	The rates of suicide attempts were highest for chronic migraine with aura (ideation 47.2%; attempts 13.9%) and lowest for migraine-free controls (2.8%) Migraine aura and depression were associated with higher risks of suicidal ideation and suicide attempts in patients with migraine. Suicide attempts with aura (OR = 5.8; 95% CI: 1.57–21.47)	I = 0 II = 1 III = 2 IV = 0 V = 1 Total score = 4
Osmophobia and allodynia are critical factors for suicidality in patients with migraine Park SP Republic of Korea	2015	*n =* 220 Age 40.3 (*SD* 13.2) (range 16–73)	Cross-sectional study Patients with migraine (with or without aura) were consecutively recruited from the headache clinic at the Department of Neurology at Kyungpook National University Hospital Patients were asked if they experienced photophobia, phonophobia, osmophobia, and allodynia during migraine attack The Mini International Neuropsychiatric Interview was used to diagnose current major depressive disorder, current generalized anxiety disorder, and suicidality The study duration was not specified	A trained neurologist diagnosed migraine based on the International Classification of Headache Disorders, 3rd edition, beta version (ICHD-3 beta)	Patients with suicidality were more likely to have chronic migraines than those without suicidality Osmophobia (Beta 0.314, adjusted OR [AOR] 3.12; 95% CI: 1.57–6.21) and allodynia (Beta 0.211, adjusted OR [AOR] 2.72; 95% CI: 1.19–6.21) were found to be critical risk factors for suicidality in patients with migraine, after controlling for depression, anxiety, and chronic migraine	I = 0 II = 1 III = 1 IV = 0 V = 1 Total score = 3
J. Aggression and its association with suicidality in migraine patients: a case-control study Park SP Republic of Korea	2018	*n =* 144 Age 37.5 (*SD* 13.2) (range 20–64)	Prospective cohort study The study enrolled 144 migraine patients who were attending the headache clinic for their first visit The patients completed various questionnaires including an Aggression Questionnaire (AQ) A trained neuropsychologist employed The Mini International Neuropsychiatric Interview Plus Version 5.0.0 (MINI) to identify suicidality The degree of aggression in migraine patients was compared to the degree of aggression in healthy controls January 2017 to September 2017	Self-reported questionnaires that were used in this study included: Aggression Questionnaire (AQ) Migraine Disability Assessment Scale (MIDAS) Patient Health Questionnaire-9 (PHQ-9) Generalized Anxiety Disorder-7 (GAD-7) Epworth Sleepiness Scale (ESS) Insomnia Severity Index (ISI)	The overall AQ score and anger and hostility subscale scores were higher in migraine patients than control patients Migraine patient's overall AQ score = 48.9 ± 12.6 (vs. healthy controls 45.8 ± 8.5) Migraine patient's anger score = 11.6 ± 4.0 (vs. healthy controls 10.2 ± 2.6) Migraine patient's hostility score = 13.7 ± 5.2 (vs. healthy controls 12.2 ± 2.7)	I = 0 II = 1 III = 1 IV = 0 V = 1 Total score = 3

* Quality ratings reported have a maximum score of 6. The criteria used to assess quality are:

(I) Representativeness of the sample to the general population: 0 points = not representative; 1 point = representative.

(II) Presence of a control/comparison group: 0 points = no control group; 1 point = control group.

(III) Number of participants with the condition (migraine): 0 points = <100; 1 point = between 101 and 500; 2 points = >501.

(IV) Longitudinal (follow-up): 0 points = no follow up; 1 point = with a follow-up.

(V) Data presentation: 0 points = unclear data presentation; 1 point = clear data presentation.

*Reproduced with permission from the work of Pompili et al. (15) and Nović et al. (8)*.

### Assessment of Methodological Quality

Two reviewers appraised the quality of citations (MB, CB). Any disagreement that arose over a specific citation were resolved by a third reviewer (LK).

### Critical Appraisal of the Individual Studies

The reviewers critically appraised the eligible articles using the JBI Critical Appraisal checklist (16) and assigned a quality of the evidence ranking (GRADE) (17). A judgment of yes, no, unclear, or not applicable was assigned to individual study elements (16). Article quality was ranked based on the GRADE assessment principles (17). Tables 2, 3 outlines the critical appraisal assessment (16) and quality of evidence (GRADE) (17) ranking of the included studies.

**Table 2:** JBI Critical appraisal of included cohort studies (16) and quality of the evidence (GRADE) (17).

**Study**	**Similar groups recruited from same population**	**Exposures measured similarly for both exposed and unexposed groups**	**Valid and reliable measurement of exposure**	**Confounding factors identified**	**Strategies to address confounding factors are stated**	**Participants were free of the outcome at the start of the study**
A. Suicidal ideation in persons with neurological conditions: prevalence, associations and validation of the PHQ-9 for suicidal ideation Altura et al. (18) Prospective cohort study	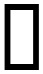	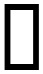	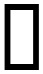	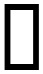	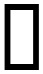	Not applicable
B. Association between lifetime headache and history of suicide attempts in the elderly Calati et al. (19) Prospective cohort study	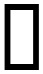	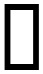	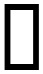	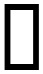	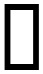	Not applicable
C. Aggression and its association with suicidality in migraine patients: a case-control study Park et al. (20) Prospective cohort study	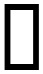	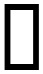	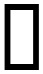	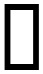	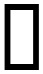	Not applicable
A. Suicidal ideation in persons with neurological conditions: prevalence, associations and validation of the PHQ-9 for suicidal ideation Altura et al. (18) Prospective cohort study	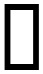	Not applicable	Not applicable	Not applicable	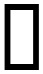	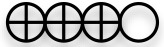 Moderate^a^
B. Association between lifetime headache and history of suicide attempts in the elderly Calati et al. (19) Prospective cohort study	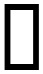	Not applicable	Not applicable	Not applicable	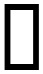	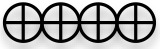 High
C. Aggression and its association with suicidality in migraine patients: a case-control study Park et al. (20) Prospective cohort study	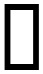	Not applicable	Not applicable	Not applicable	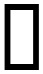	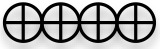 High

a *Risk of bias sufficient to downgrade one level*.

**Table 3:** JBI Critical appraisal of included cross-sectional studies (16) and quality of the evidence (GRADE) (17).

**Study**	**Clearly defined sample inclusion criteria**	**Detailed description of study participants and setting**	**Valid and reliable measurement of exposure**	**Objective and standard criteria used for condition measurement**	**Confounding factors identified**	**Strategies to address confounding factors are stated**	**Appropriate statistical analysis performed**	**Quality of the evidence (GRADE)**
D. Association between migraine and suicidal behavior among Ethiopian adults Berhane et al. (25) Cross-sectional study	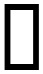	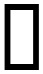	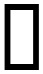	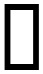	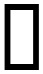	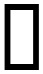	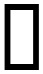	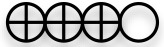 Moderate^b^
E. Association of migraine headaches with suicidal ideation among pregnant women in Lima, Peru Friedman, et al. (26) Cross-sectional study	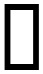	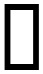	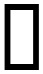	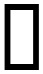	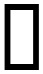	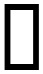	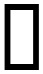	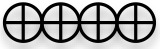 High
F. Association between migraine and suicidal behaviors: a nationwide study in the USA Friedman et al. (27) Cross-sectional study	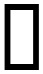	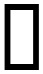	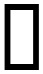	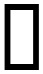	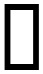	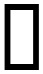	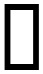	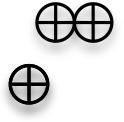 High
G. Suicide attempts among those with migraine: findings from a nationally representative Canadian study Fuller-Thomson et al. (21) Cross-sectional study	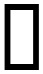	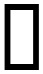	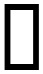	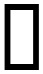	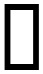	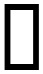	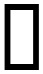	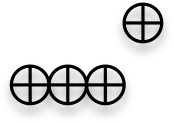 High
H. Risk and predisposing factors for suicide attempts in patients with migraine and status migrainosus: A nationwide population-based study Harnod et al. (22) Cross-sectional study	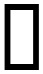	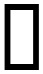	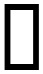	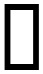	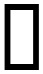	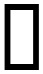	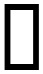	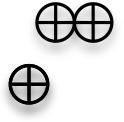 High
I. Association of suicide risk with headache frequency among migraine patients with and without aura Lin et al. (23) Cross-sectional study	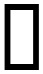	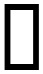	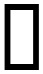	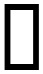	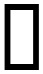	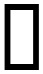	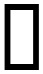	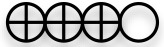 Moderate^c^
J. Osmophobia and allodynia are critical factors for suicidality in patients with migraine Park et al. (24) Cross-sectional study	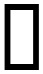	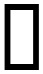	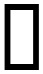	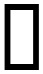	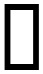	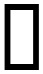	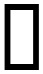	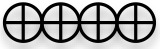 High

b Risk of bias sufficient to downgrade one level.

c *Risk of bias and inconsistencies sufficient to downgrade one level*.

### Quality Scores

The studies were assessed and assigned a quality score adapted from Pompili et al. (15) and Nović et al. (8). Allocated quality scores considered five study features such as the sample representativeness, which included a comparison group, the number of participants with migraine, whether follow-up was performed, the presence of a longitudinal study, and the clarity of presented data. The quality scores of the articles included in this review are outlined in Tables 2, 3.

### Data Analysis

Extracted data was combined to determine the overall effect for each study design where possible. Information related to risk factors from the included studies such as participant age, study design, and characteristics are described in Table 1.

## Results

### Study Selection

The database search yielded a total of 542 citations. An additional nine references were identified through other sources. After the removal of 69 duplicates, 482 articles were included for eligibility assessment. After title and abstract screening 461 articles were excluded. The remaining 21 articles (listed below) were independently assessed for eligibility based on the full text review inclusion and exclusion principles. Eleven studies were excluded due to their interest in alternative outcomes (*n* = 5) and study design (*n* = 6). Ten articles were included in this systematic review. The results for article selection are presented in the PRISMA chart in Figure 1.

### Study Characteristics

The articles included were published between January 2015 and November 2019. Two of the studies were conducted in Canada (18, 21), two studies in Taiwan (22, 23), two in the Republic of Korea (20, 24), and one study each in Ethiopia (25), France (19), Peru (26), and the USA (27). Seven of the studies used a cross-sectional study design (21–27) and three were prospective cohort studies (18–20).

Participants were recruited from outpatient clinics in six of the studies (18, 20, 23–26) and from nationwide samples in three studies (21, 22, 27). Clinic populations were recruited from the following outpatient clinics:

- Epilepsy (18)
- Multiple Sclerosis (18)
- Stroke (18)
- Internal medicine (25)
- General surgery (25)
- Gynaecology (25)
- Prenatal care (26)
- Headache (18, 20, 23, 24).

The nationwide samples were selected from hospital billing data (27), community-based mental health survey data (21), and a national health database (22). A single study used population-based samples of participants from the electoral role of the Montpellier district (19).

The number of participants diagnosed with migraine was <100 in one study (19) and between 100 and 500 in four studies (18, 20, 24, 26). Whilst the sample population was over 500 in five of the studies (21–23, 25, 27), two of those reported limitations regarding generalizability (23, 25). Berhane et al. (25) and Lin et al. (23) focused on a hospital-based population. The remaining three studies used a large sample size that was nationally representative of the population (21, 22, 27). Longitudinal follow up was not conducted in any of the studies. All of the studies included a control or comparison group and presented their data clearly.

### Risk of Bias Within Studies

The Cochrane Risk of Bias comparison tool (28) was used to assess internal validity based on key criteria with each rated as high, low, or unclear. Two independent reviewers evaluated the quality of the identified citations. The majority of the studies were appraised as having a low risk of detection bias with the exception of two studies that were assessed as high risk (21, 26). The risk of performance bias was assessed as low for three of the studies (22, 24, 27). Other sources of bias were appraised as high risk for eight of the studies (18–21, 23, 25–27). These included:

- Social desirability bias and underreporting (18, 20, 21, 23, 25, 27)
- Recall bias (18–20, 23, 25, 26)
- Bias introduced by sample pooling for multivariate analysis (19, 23)
- Bias introduced by focusing on specific age groups, for example, the elderly (19)
- Coding and data errors (27).

A low risk rating was allocated for reporting bias and attrition bias in all studies. Overall, 20% of the included studies (22, 24) were assessed as high quality associated with less risk of bias, 60% were of moderate quality (18–20, 25–27), and 20% were between moderate and low quality (21, 23). The ratings of all included studies are shown in Table 1.

### Diagnosis and Assessment of Migraine

Diagnosis and assessment of migraine varied between the studies. Altura et al. (18) did not discuss diagnosis and assessment of migraine. The primary aim of this study was to validate the Patient Health Questionnaire (PHQ)-9 (18, 29). It was assumed that patients had a pre-existing diagnosis of migraine as they were recruited from an outpatient headache clinic.

Four studies of migraine applied the International Classification of Headache Disorders-2 or 3 diagnostic criteria (ICHD-II or III criteria) (2, 23–26). Berhane et al. (25) employed a trained nurse to administer a structured migraine questionnaire to classify migraine disorder based on the ICHD-II criteria (25). Similarly, Friedman et al. (26) used trained interviewers to classify migraine by administering a questionnaire during early pregnancy, including migraine and probable migraine, based on the ICHD-III beta criteria. Lin et al. (23) participants were identified as with aura or without aura based on the ICHD principles (2). Park et al. (24) reported migraine diagnosed by a trained neurologist based on the ICHC-III beta criteria (2). Two studies used the International Classification of Diseases, 9th Revision, Classification Modification (ICD-9-M) (22, 27, 30) diagnosis codes to diagnose migraine.

In addition, Harnod et al. (22) analyzed the data according to diagnosis codes for different cohorts including regular migraine and status migrainosus (31). In one study, a neurologist assessed headache cases based on the International Headache Society guidelines (2, 19) and Park et al. (20) enrolled patients who were attending a headache clinic for their first visit and used the self-report Migraine Disability Assessment Scale (MIDAS) (32) to determine a diagnosis of migraine.

Three of the studies did not specify migraine types and reported on the presence of migraine only (18, 21, 22). Altura et al. (18) recruited patients from a headache clinic whilst Fuller-Thomson et al. (21) included patients with migraine that had being occurring for 6 months or more. Harnod et al. (22) differentiated regular migraine from status migrainosus. The remaining seven studies categorized migraine types and symptoms to varying degrees. Migraine types were specified in three studies (19, 26, 27). Calati et al. (19) included both migrainosus and non-migrainous lifetime headache types whilst Friedman et al. (26) classified migraine and probable migraine in pregnant women. In addition to migraine, Friedman et al. (27) included other headache types according to the ICD-9-CM diagnosis codes (30) for tension headaches and headache. Four studies analyzed subtypes of migraine and associated migraine symptoms (20, 23–25). Berhane et al. (25) defined frequency and pain characteristics and common symptoms. Lin et al. (23) investigated migraine with or without aura, categorized migraine frequency as chronic, high, medium, and low, and also included a control group with no history of migraine in their family. Park et al. (24) considered episodic and chronic migraine with or without aura and associated symptoms whilst also reporting on medication overuse headaches and high headache intensity. Park et al. (20) also investigated these subtypes with the exception of the presence of migraine with or without aura.

### Diagnosis and Assessment of Suicidal Behaviors

Diagnosis and assessment of suicidal behaviors also varied between the studies. Three studies used the Mini-International Neuropsychiatry Interview (MINI) (33), a Diagnostic and Statistical Manual of Mental Disorders (DSMV-IV) (34) criteria to identify suicidal behaviors (19, 20, 24). In all three studies, a trained interviewer (either a nurse, psychologist, or neuropsychologist) administered the MINI to identify suicidality (19, 20, 24). In addition, Calati et al. (19) referred positive cases to a panel of three psychologists for review. Whilst Calati et al. (19) employed the MINI to identify suicide attempts only, Park et al. (20, 24) examined suicide attempts, suicidal ideation, and suicide plans.

Two studies (22, 27) identified suicidal behaviors based on the ICD-9-M (30) diagnosis. Harnod et al. (22) examined suicide attempts only whilst Friedman et al. (27) identified suicidal ideation, suicide attempts, and was the only study in this review to include self-inflicted injury. In two studies, trained health professionals used the Semi Structured Composite International Diagnostic (SCID) and interviewed participants to identify suicidal ideation, suicide plans, and suicide attempts (18, 25, 34). Berhane et al. (25) used the Composite International Diagnostic interview (CIDI) (35) to evaluate depression as well as self-reported suicidal behaviors that were identified as ideation, plans, and attempts. Friedman et al. (26) used the Patient Health Questionnaire-9 (PHQ-9) (29) and assessed suicidal ideation. Two studies examined responses to survey questions to identify suicide attempts (21, 23) and suicidal ideation (23).

### Comorbidities and Other Associated Conditions

All of the studies included other comorbidities and conditions that were mainly associated with mental health. Nine studies included anxiety (18–25, 27) and depression or major depressive disorder (18, 19, 21–27). Four studies in total investigated substance abuse and dependence related to either alcohol (19, 21, 22, 27) or drugs and other substances (21). Three studies investigated participant responses to questions about sleep quality and insomnia (19, 22, 23). Three studies also examined other psychiatric conditions including psychosis, manic and hypomanic episodes, schizophrenia, post-traumatic stress disorder, and childhood and adolescent trauma (19, 22, 27). In addition, Calati et al. (19) collated data on risk factors associated with hypertension, hypercholesterolemia, and diabetes. Friedman et al. (26) included pregnant participants in their study, whilst Park et al. (20) examined the degree of aggression (physical and verbal aggression, anger, and hostility) among migraineurs compared to healthy participants (20). Finally, only one study assessed chronic pain and suicide attempts (21).

### Migraine and Suicidal Ideation

Two studies analyzed the migraine and suicidal ideation link only (18, 26) whilst a third study also included suicide attempts (23). Altura et al. (18) indicated that the prevalence of suicidal ideation in migraineurs was higher than that reported in general populations. The factors related with suicidal ideation included depression, migraine, and anxiety. Similarly, Friedman et al. (26) reported that, after adjusting for some confounders, pregnant women with migraine showed almost a two times higher incidence of suicidal ideation. Similarly, women who were experiencing both migraine and depression showed an almost four times higher rate of suicidal ideation compared to those who didn't have any of those disorders. (26) Lin et al. (23) observed that migraine aura and depression were correlated with suicidal ideation and suicide attempts (23). Migraine aura and depression severity projected suicidal ideation of migraineurs, especially with chronic migraine with aura (23).

### Migraine and Suicide Attempt

Three studies assessed the link between migraine and suicide attempt (19, 21, 22). Interestingly, all three studies recruited participants from the general population. Whilst Fuller-Thomson et al. (21) and Harnod et al. (22) examined nationwide population data of Canada and Taiwan, Calati et al. (19) study participants were community-dwelling individuals recruited out of the 15 electoral rolls of the Montpellier district in France and were aged 65 years and above (19). Calati et al. (19) reported that lifetime headache (both migraine and non-migraine) was associated with lifetime suicide attempts. Fuller-Thomson et al. (21) revealed that individuals with migraine had an almost 3-fold higher risk of attempting suicide compared with non-migraineurs. Harnod et al. (22) reported that patients who experienced status migrainosus had around a twice higher likelihood of suicidal attempts in comparison to the control group (22). Suicide attempts were higher in participants with depression, anxiety, insomnia, and alcohol-related illnesses (22).

### Migraine and Suicidal Behaviors

Three studies analyzed the relationship of migraine with a range of suicidal behaviors (20, 24, 25). A single study (27) also included self-inflicted injury in their analysis. Friedman et al. (27) analyzed a national cohort of hospitalizations in the USA (27) and reported on migraineurs with depression, anxiety, or post-traumatic stress disorder (PTSD) (27). They reported that individuals with migraine had around twice the likelihood of suicidal behaviors (including self-inflicted injury) compared with non-migraineurs (OR = 2.17; 95% CI: 1.80–2.61) (27). Friedman et al. (27) performed separate analyses and found that migraine was linked with some psychiatric disorders, such as anxiety, depression, and PTSD, and could lead to higher chances of suicidal behaviors (27). Park et al. (24) recruited patients from a headache clinic in a hospital (24) and found that patients with suicidality were about three times more likely to have chronic migraines than those without suicidality.

Berhane et al. (25) noted that migraine and suicidal behaviors are highly correlated even after adjusting for some confounders (such as substance use and socio-demographic factors) (25). The reported rates of suicidal ideation, suicide plans, and suicide attempts were consistently higher in the migraine cohort compared with non-migraineurs (25). Berhane et al. (25) reported that after stratifying by history of depression, the odds of suicidal behavior was twice as high amongst migraineurs than non-migraineurs (25).

Park et al. (24) also reported that osmophobia (or olfactophobia that refers to a fear, aversion, or psychological hypersensitivity to odors) and allodynia (“refers to central pain sensitization increased response of neurons following normally non-painful, often repetitive, stimulation”) (24) were found to be critical predictors of suicide after adjusting for depression, anxiety, and chronic migraine (OR = 3.12; 95% CI: 1.57–6.21 and OR = 2.72; 95% CI: 1.19–6.21, in order) (24). In a second study, Park et al. (20) enrolled 144 migraine patients to the study. The suicide rate was higher among chronic migraine patients (42.9%) compared with episodic migraine patients (12.5%) (20). The patients completed various questionnaires, including an Aggression Questionnaire (AQ) (36). Those suffering from migraine compared with the control group (20) showed higher anger, hostility, and overall scores.

## Discussion

The aim of this review was to systematically examine the likelihood of suicidal behaviors such as suicidal ideation, suicide attempts, suicide plans, and self-harm or self-inflicted injury among populations of migraine patients older than age 16 years. Adolescent and adult migraineurs were chosen for this systematic review, given that migraine is recognized as the most prevalent and debilitating headache types (in terms of hospital and clinic visits) and the increase in prevalence among young adults makes it one of the major causes of disability among working age adults (7). Similarly, suicide among children is rare while the highest rates are observed in middle age. The studies that achieved a low bias rating overwhelmingly support a strong relationship between migraine and suicidal behaviors, as have earlier publications.

The observed trend of a strong correlation between migraine and suicidal ideation observed after adjusting for confounders by Friedmann et al. (9) was reflected in five of the articles reviewed in this study (19, 21–23, 26). Calati et al. (19) reported a strong link between lifetime suicide attempts and lifetime headache in an elderly sample population after adjusting for confounding variables such as depression. Friedman et al. (26) also noted that pregnant women with migraines in Peru had a higher rate of suicidal ideation after adjusting for depression and other confounders. Harnod et al. (22) controlled for most psychiatric comorbidities in their analysis and found that suicide attempts among patients with status migrainosus (22) and specific psychiatric comorbidities was high (22). Lin et al. (23) also reported that migraine aura and depression severity were predictors of suicidal ideation among migraineurs (23) after adjusting for possible confounding factors.

Aly et al. in their study of migraine and the risk of suicide highlighted the fact that migraine and depression are common comorbid conditions and that both episodic and chronic migraine have been associated with comorbid psychiatric conditions such as depression. Previous systematic reviews have explored this relationship, as did all of the articles reported in this review (8, 9). Friedman et al. (26) also discussed the association between environmental risk factors, migraine, depression, and suicidal behaviors. Fuller-Thomson et al. (21) provided an additional dimension to their research and highlighted a number of possible risk factors, such as adverse childhood events that might predispose migraineurs to suicidal attempts (21). Fuller-Thomson et al. (21) also found that patients who had witnessed or experienced domestic violence demonstrated higher rates of suicidal attempts compared with those who had not experienced any adverse childhood events, whether or not they were migraineurs. In addition, they suggested that the bi-directional relationship between migraine and depression might also be extended to include other variables such as drug and alcohol abuse (21).

Lin et al. (23) examined the relationship of migraine with depression and other comorbidities such as anxiety and sleep quality (23). Park et al. (24) focused on the association of sensory hypersensitivities and suicidality in migraineurs and did not examine the effects of comorbid diseases. They excluded patients with serious medical, neurological, and psychiatric disorders from their study (24). They reported that osmophobia and allodynia are as significant as psychiatric disorders (24) in the determination of suicidality in patients with migraine (24). In a different study, Park et al. (20) focused on an area of minimal research to date and studied aggression and suicidality in migraine patients. They found higher rates of aggressive behaviors among chronic migraine patients (20) Friedman et al. (9) reported on the association of anxiety, depression, and anger with headache triggers, intensity of headache pain, and response to treatment.

Studies previously reviewed by Nović et al. (8) have already suggested that the severity, frequency, and intensity of migraine pain influences the risk of suicidal behavior in migraineurs, with some authors suggesting that pain might be an independent risk factor. Four studies in this review have also discussed this association (19, 22, 23, 25). Calati et al. (19) recommended further investigation of suicidality and the role of pain chronicity and severity. The comorbidity of suicidal tendencies and migraineur pain severity was also highlighted by Berhane et al. (25) and Harnod et al. (22) who revealed that suicide attempts increased after 5 years following status migrainosus diagnosis (22) and considered that the severity and duration of pain might play a critical role.

Nović et al. (8) commented on the limitation among retrieved studies related to the variability in the classification of migraine and its subtypes within the studies. Some studies looked at migraine as a whole while others specified subtypes. They noted that some studies did not differentiate among migraine types, such as with aura or without aura (8). The systematic review revealed that migraine with aura shows a stronger relationship with suicidal behavior than migraine without aura, and this remains evident even after controlling for other factors such as age, gender, and psychiatric conditions, suggesting an independent association between migraine with aura and suicidal behaviors (8). This finding was supported by Friedman et al. (9) who stated that migraine with aura is consistently more strongly associated with suicide ideation compared with migraine without aura. Similarly, this review revealed that studies varied in their investigation and analysis of headache subtypes. Calati et al. (19) were unable to investigate migraine and non-migraine headache subtypes as the lifetime suicide attempt sample was too small for analysis. In addition, they focused on lifetime suicide attempts only and recommended that further studies should investigate different suicidal phenotypes including suicide, suicidal ideation, and self-harm (19).

Friedman et al. (26) recommended that further studies investigate the relationship between migraine phenotypes with suicidal ideation. Similarly, Friedman et al. (27) could not distinguish between migraine subtypes with or without aura as they analyzed hospital diagnosis codes and found that there was a coding error for migraine subtypes (27). Fuller-Thomson et al. (21) investigated data from a population-based sample that did not allow for differentiation between migraine subtypes such as chronic and episodic (21), with aura or without aura, and degree of severity (21). In addition, age of onset and timing of suicide attempts was unreported in the data set and self-reported migraine could not be validated (21). Harnod et al. (22) differentiated between migraine and status migrainosus only whilst Lin et al. (23) examined migraine with aura and without aura and found that migraine with aura was a strong risk factor of suicidal ideation/attempt in the clinic-based population (23). Park et al. (24) did not find such results and noted that this finding might have been because the hospital-based population had a low number of migraine patients with aura (*n* = 17) and the cohort age range was wide compared with other studies that identified the association with aura.

Nović et al. (8) noted that there were limitations in the variations of the way suicidality was measured. This review also revealed variations in data collection and measurement. Altura et al. (18) conducted telephone interviews and acknowledged that PHQ-9 questionnaires were not always completed on the same day as the SCID interview. Friedman et al. (27) based their findings on ICD-9 hospital diagnosis codes that did not differentiate between suicidal ideation, suicide attempt, and non-suicidal self-inflicted harm (27). The authors reported that this might introduce the potential error of misclassifications of suicidal behaviors (27). Fuller-Thomson et al. (21) acknowledged that their study used a crude measure to assess pain that minimized the ability to identify those with the most severe pain. In addition, only one self-report question assessed suicide attempt (21). Harnod et al. (22) cited the possible miscoding and under-diagnosis of suicide events in the database as a potential study limitation in addition to restrictions around obtaining further information, as patients were anonymized and therefore not contactable.

Nović et al. (8) discussed the shared biological mechanisms for migraine, suicidal behavior, and major affective disorders. Similarly, Friedman et al. (9) reported that migraine, major depressive disorder, and suicidal ideation may be influenced by both genetic and environmental factors including stressful life events that affect the neurobiological systems. Furthermore, serotonin transported polymorphisms have been associated with the frequency of migraine attacks, depressive symptoms, and suicidal behaviors (9). Aly et al. (1) provided the opinion that genetics are partially responsible for the risk of suicide and reduced serotonergic activity is linked with suicidal behavior. Whilst the evidence is limited, six of the studies in this review discussed the connection between biological mechanisms and migraine, psychiatric disorders, and suicidal behaviors (19, 21–23, 25, 26). Two studies discussed psychosocial and environmental influences (19, 26) and five studies explored the role of genetic variability (19, 21–23, 25, 26). Two of the studies discussed the cultural and ethnic differences between Asian and Western populations (22, 24). Harnod et al. (22) reported that these distinctions be for the difference between their Taiwanese study and other studies carried on in Western societies (24).

Given that the results of this study support the fact that people with migraine are at an increased risk of suicide, they are also more likely to experience anxiety which is considered as a strong risk factor for suicide (Sareen, 2011). Therapeutic strategies should consider screening for anxiety, suicidal behavior or ideation, and other psychiatric disorders. The treatment strategies targeting both migraine and comorbid disorders will have a better outcome for the migraine sufferers and could prevent more serious actions such as suicide.

Based on a rigorous recent metanalysis, the communication of suicidal intentions occurs in almost half of people who decide to end their life by suicide (37). Thus, promoting better care for migraine is the first step in preventing suicidal behaviors among patients with migraine. Despite being a common neurological disorder in the world, affecting one in seven people worldwide, migraine continues to be underrecognized, underdiagnosed, and undertreated (7, 38).

Despite this sad truth, migraine continues to be worst managed medical disorder worldwide, resulting in the first ever global campaign on migraine, the “painful truth” during World Brain Day 2019 (7).

It is critical to recognize and promote the global, regional, and local interest of people with migraine. All patients with migraine should have access to appropriate medical care. All health care professionals including physicians, nurses, and psychologists should have access to adequate and up to date training in migraine, associated comorbidities, and management as a matter of priority (38).

Nović et al. (8) found that the international representativeness of the studies retrieved for the systematic review was limited. Thus, this review retrieved more recent studies from Peru, Ethiopia, Canada, and Taiwan, as well as the US, though five of these studies used clinic-based patients and therefore the results may only be generalizable to worldwide hospitalized populations (18, 20, 23–25). The two studies conducted by Friedman et al. (26, 27) both assessed large samples, though one study focused on pregnant women only (26) and the other was a nationally representative sample of American adult hospital inpatients and again warrants caution when generalizing to community-based patients (27). The large representative data sets of Fuller-Thompson et al. (21) and Harnod et al. (22) must also be seen as study strengths (21, 22).

Underreporting in the form of recall bias was reported in three studies (19, 25, 26) and social desirability bias was reported in two others (25, 27), while Harnod et al. (22) acknowledged underestimation bias in their report due to the exclusion of a number of factors.

This review is also limited in consideration of only late adolescents and adult cohorts and the design methodology used in the retrieved English language studies. Seven of the studies used a cross-sectional study design (21–27), thereby limiting conclusions based on causality, and not surprisingly leading to a recommendation of future longitudinal studies and population-based research. Nović et al. (8) also highlighted these limitations several years ago. Further studies should broaden the populations to be examined to include younger adolescents and people from other cultural cohorts. Consideration of other prevalent headaches types such as tension headaches and their relationship to suicidal behaviors or other psychiatric disorders also remains necessary.

In all studies, the clinical implications of migraine as such a common underrecognized, underdiagnosed, undertreated (7, 38), and poorly managed neurological disorder affecting one in seven people worldwide, have been highlighted. Whether previous systematic reviews (8, 9) or the expert opinion of Aly et al. (1), all recommend screening and early identification of suicidal behaviors and psychiatric comorbidities in at-risk migraineurs (1, 9, 18–27). Collectively, all the studies in this review recognize and promote the need for enhanced medical care of people with migraine and on-going training for health care professionals in migraine, associated comorbidities, and management (8) as a matter of priority (38).

## Conclusion

Migraine is often associated with lifetime disability and negative quality of life. This review has investigated the association between migraine and suicidal behaviors as reported in ten recent international studies. Collectively, all the studies suggest an association between migraine, suicidal behaviors, and comorbidities, including psychiatric disorders, and demonstrate an increased risk of suicidal behaviors in both clinical and general population migraineurs.

Future studies are needed to facilitate the development of clinical guidelines for risk assessment, targeted interventions, and evidence-based treatment of migraine to minimize the risk of suicide among this vulnerable population.

## Author Contributions

LK, CB, and MB carried out the database search, screening, quality assessment, data extraction, and analysis. LK and DH wrote the first draft of the manuscript. TW and SC revised the initial drafts and gave scientific contribution. All authors provided critical feedback, helped shape the research, analysis, manuscript, and contributed to the conceptualization and design of the research.

## Conflict of Interest

The authors declare that the research was conducted in the absence of any commercial or financial relationships that could be construed as a potential conflict of interest.
